# National trends and disparities in mortality involving bladder cancer and substance use disorders in the United States, 1999–2023

**DOI:** 10.3389/fonc.2026.1883338

**Published:** 2026-09-29

**Authors:** Kanza Atif, Unsa Arif, Muhammad Ali Gorish, Safa Mohammed Saleem, Aleena Ihtasham, Huzafa Ali, Fatima Sohail, Shan e Ali Shoukat, Taha Alam, Simran Parkash, Muhammad Hanif Khan, Muhammad Atif Mazhar

**Affiliations:** 1 College of Medicine, Alfaisal University, Riyadh, Saudi Arabia; 2 Continental Medical College, Lahore, Punjab, Pakistan; 3 Services Hospital Lahore, Lahore, Pakistan; 4 Tbilisi State Medical University, Tbilisi, Georgia; 5 King Edward Medical University, Lahore, Pakistan; 6 Combined Military Hospital (CMH) Multan Institute of Medical Sciences, Multan, Pakistan; 7 Jinnah Sindh Medical University, Karachi, Sindh, Pakistan; 8 Dow University of Health Sciences, Karachi, Sindh, Pakistan; 9 Spinghar University, Kabul, Afghanistan

**Keywords:** age-adjusted mortality rate (AAMR), bladder cancer (BC), CDC WONDER Database, mortality trends, racial disparities, substance use disorder (SUD), United States

## Abstract

**Background:**

Although bladder cancer mortality has modestly declined in the United States, co-occurring substance use disorders (SUDs) may contribute to adverse outcomes and widening health disparities among affected individuals. This study evaluated temporal trends in mortality involving both bladder cancer and SUDs in the United States between 1999 and 2023.

**Methods:**

Mortality data related to patients aged ≥25 were obtained from the CDC WONDER Database. Deaths involving bladder cancer were identified using ICD-10 code C67 in combination with ICD-10 codes F10–F19 for substance use disorders listed among multiple causes of death. Age-adjusted mortality rates (AAMRs) per 100,000 population were calculated and Joinpoint regression was used to estimate annual percent changes (APCs) and average annual percent changes (AAPCs).

**Results:**

Between 1999 and 2023, 63,258 deaths involving both bladder cancer and SUDs were identified. Overall, the AAMR increased significantly at an annual average percentage change (AAPC) of 13.38% (95% CI: 10.69–16.14). Males had a substantially higher mortality burden than females (50,299 vs 12,959). White individuals and adults aged ≥85 were accounted for the largest mortality burden. Mortality rates were consistently higher in nonmetropolitan populations and increased across all U.S. census regions.

**Conclusion:**

Mortality involving bladder cancer and coexisting SUDs increased over time and mirrored broader national substance-related mortality trends. These findings support integrated oncology- and addiction-focused care approaches, as well as public health strategies aimed at improving access to cancer care, addiction treatment, and rural health services.

## 1 Introduction

Bladder cancer makes up about 4.6% of all newly diagnosed cancers and contributes to 2.8% of cancer-directed deaths in the US, which is the second most frequent genitourinary malignancy and the sixth most common cancer (1). 70% of bladder cancers are classified as localized or non-muscle-invasive bladder cancer (NMIBC), whereas the remaining 30% are muscle-invasive bladder cancer (MIBC) at the time of diagnosis (2). Men experience substantially higher overall bladder cancer incidence and mortality, whereas women, particularly Black women, have been reported to experience poorer stage-specific survival and worse cancer-specific outcomes, largely attributable to delayed diagnosis, differences in healthcare access, and more advanced disease at presentation (1). Substance use disorders (SUDs) are increasingly recognized as an important comorbidity among cancer survivors, particularly in the context of chronic pain management, opioid exposure, and psychosocial stress factors associated with cancer care. A hospital-based case-control study reported an approximately six-fold increased risk of bladder cancer among heroin/opium users (3). Cannabis exposure had also been investigated as a potential risk factor for bladder cancer development (4). Coexisting SUDs may complicate the management of bladder cancer because of challenges related to pain control, medication safety, and treatment adherence (5).

Despite growing recognition of the intersection between cancer care and substance use, national mortality patterns involving bladder cancer and co-occurring SUDs remain poorly characterized. Existing literature has primarily focused on either bladder cancer mortality trends or substance-related mortality independently, with limited attention to their overlap at the population level. Understanding these patterns is important for identifying vulnerable populations, informing integrated oncology-addiction care strategies, and addressing widening public health disparities. To address this knowledge gap, we analyzed national mortality data from the CDC WONDER Multiple Cause of Death database from 1999 to 2023 to evaluate temporal trends and demographic disparities in mortality involving both bladder cancer and SUDs in the United States.

## 2 Methods

### 2.1 Study setting and population

Mortality data involving both bladder cancer and substance use disorders (SUDs) in the U.S population aged ≥25 years were retrieved from the CDC WONDER Multiple Cause-of-Death (MCD) database and analyzed for the period from January 1999 to December 2023. Deaths were included when both bladder cancer (ICD-10 C67) and Substance use disorders were identified using ICD-10 codes F10–F19, including disorders related to alcohol (F10), opioids (F11), cannabinoids (F12), sedatives or hypnotics (F13), cocaine (F14), other stimulants including caffeine (F15), hallucinogens (F16), tobacco (F17), volatile solvents (F18), and multiple or other psychoactive substances (F19) were listed anywhere on the death certificate, either as the underlying or contributing cause of death. Similar ICD-10 coding strategies have been used in prior CDC WONDER mortality analyses (6, 7). IRB approval wasn’t required as a publicly available dataset was used for this study. The reporting of this study is in accordance with Strengthening the Reporting of Observational Studies in Epidemiology (STROBE) guidelines (8).

### 2.2 Data abstraction

Demographic and geographic variables extracted from CDC WONDER included year of death, sex, age group, race/ethnicity, census region, urbanization status, place of death, and state-level mortality data. Place of death categories included death at healthcare settings, like medical facilities (outpatient, emergency room, inpatient, death on arrival, or unknown status), home, hospice, and nursing home/long-term care facilities, and unknown locations. Race/ethnicity categories included non-Hispanic (NH) White, NH Black or African American, Hispanic or Latino, NH American Indian or Alaska Native, and NH Asian or Pacific Islander. For detailed descriptive analyses, the NH Asian or Pacific Islander category was further disaggregated into Asian and Native Hawaiian or Pacific Islander subcategories. Urbanization status was classified using the 2013 National Center for Health Statistics Urban-Rural Classification Scheme, categorizing counties as metropolitan or nonmetropolitan (9). Urban–rural classification data were available in the CDC WONDER database only through 2020 because the National Center for Health Statistics (NCHS) has not yet released updated urbanization classifications based on the 2020 U.S. Census. Therefore, urbanization-specific analyses were limited to the period 1999–2020. Census regions were categorized into Northeast, Midwest, South, and West according to U.S. Census Bureau definitions (10).

### 2.3 Statistical analysis

Crude mortality rates (CMRs) and age-adjusted mortality rates (AAMRs) per 100,000 population were calculated.by dividing the number of deaths in each category by the corresponding U.S. population for that year. AAMRs were age-adjusted to the 2000 U.S. standard population and expressed per 100,000 population (11). National temporal trends in AAMRs were analyzed using the Joinpoint Regression Program (Version 5.4.0.0, National Cancer Institute) (12). Log-linear regression models were fitted to the annual age-adjusted mortality rates using a grid search algorithm, with a minimum of 0 and a maximum of 4 joinpoints. The optimal model was selected using the permutation test (4,499 permutations; overall significance level = 0.05). Standard errors of the age-adjusted mortality rates were incorporated into the analysis assuming uncorrelated errors. Annual percent change (APC) and average annual percent change (AAPC) with 95% confidence intervals (CIs) were estimated using the parametric method, with AAPCs calculated over the entire study period. All statistical analyses were performed using CDC WONDER and the Joinpoint Regression Program. A two- sided P value <no><0.05</no> was considered statistically significant. Because CDC WONDER suppresses low-frequency mortality counts to preserve confidentiality, subgroup analyses involving smaller racial/ethnic populations contained limited data and potentially unstable trend estimates, which were interpreted with caution. Comparisons across demographic and geographic subgroups were descriptive in nature, and no formal statistical tests for subgroup differences or interactions were performed.

## 3 Results

Between 1999 and 2023, 63,258 deaths involving both bladder cancer and SUDs were identified in the United States (**Table 1****).**

**Table 1:** Mortality co-occurring with bladder cancer and substance use disorders stratified by sex and race among U.S. adults, 1999–2023.

Year	Overall deaths	Female	Male	American Indian or Alaska Native	Asian or Pacific Islander	Black or African American	White	Asian	Hispanic or Latino	Population (overall)
1999	186	43	143	–	–	13	169	–	–	180,408,769
2000	238	56	182	–	–	14	220	–	–	181,984,640
2001	235	52	183	–	–	0	224	–	–	184,305,128
2002	212	37	175	–	–	10	195	–	–	186,208,028
2003	839	194	645	–	–	38	776	–	18	188,090,429
2004	1,207	259	948	–	–	46	1,126	–	23	190,205,384
2005	1,614	340	1,274	10	–	75	1,493	–	28	192,551,384
2006	1,826	383	1,443	–	–	78	1,688	–	42	195,019,359
2007	2,012	430	1,582	–	11	97	1,855	–	40	197,403,777
2008	2,320	492	1,828	16	16	84	2,152	–	47	199,795,090
2009	2,396	483	1,913	–	–	103	2,232	–	41	202,107,016
2010	2,525	498	2,027	11	15	134	2,313	–	46	203,891,983
2011	2,817	595	2,222	10	15	143	2,603	–	44	206,592,936
2012	3,128	635	2,493	–	13	141	2,909	–	51	208,826,037
2013	3,166	647	2,519	11	25	135	2,933	–	57	211,085,314
2014	3,296	684	2,612	11	20	165	3,036	–	53	213,809,280
2015	3,609	744	2,865	17	20	181	3,319	–	63	216,553,817
2016	3,723	756	2,967	12	25	198	3,381	–	88	218,641,417
2017	3,778	805	2,973	21	29	206	3,422	–	95	221,447,331
2018	3,834	737	3,097	12	18	215	3,484	12	95	223,311,190
2019	4,075	844	3,231	16	16	212	3,717	14	104	224,981,167
2020	4,021	746	3,275	18	32	201	3,661	28	99	226,635,013
2021	3,987	809	3,178	–	–	216	3,607	25	103	228,238,412
2022	4,105	862	3,243	23	–	230	3,684	34	109	229,508,599
2023	4,109	828	3,281	15	–	251	3,679	27	118	231,529,762
**Total (1999–2023)**	**63,258**	**12,959**	**50,299**	**203**	**255**	**3,186**	**57,878**	**140**	**1,364**	**—**

The Asian category (n = 140) is a subset of the Asian or Pacific Islander category (n = 255) and is presented as a further descriptive breakdown; these categories should not be summed.

Bold values represent the total number of deaths recorded during the entire study period (1999–2023).

### 3.1 Annual trends

Overall mortality involving bladder cancer and SUDs increased significantly during the study period (AAPC, 13.38%; 95% CI, 10.69–16.14; p<0.0001). Mortality burden increased significantly between 1999 and 2005 (APC = 51.63%; 95% CI, 38.21–66.35; p < 0.0001). The rate of increase slowed between 2005 and 2013 (APC = 6.39%; 95% CI, 3.26–9.62; p = 0.000418) and then reached a plateau between 2013 and 2023 (APC = 0.20%; 95% CI, –1.18 to 1.61; p = 0.7615). From 0.1001 to a peak of 1.5092 per 100,000, the overall age-adjusted mortality rate increased between 1999 and 2016 before slightly declining to 1.4524 per 100,000 in 2023. (**Figure 1**, **Tables 1**, **2**, **Supplementary Table S1**).

**Figure 1:**
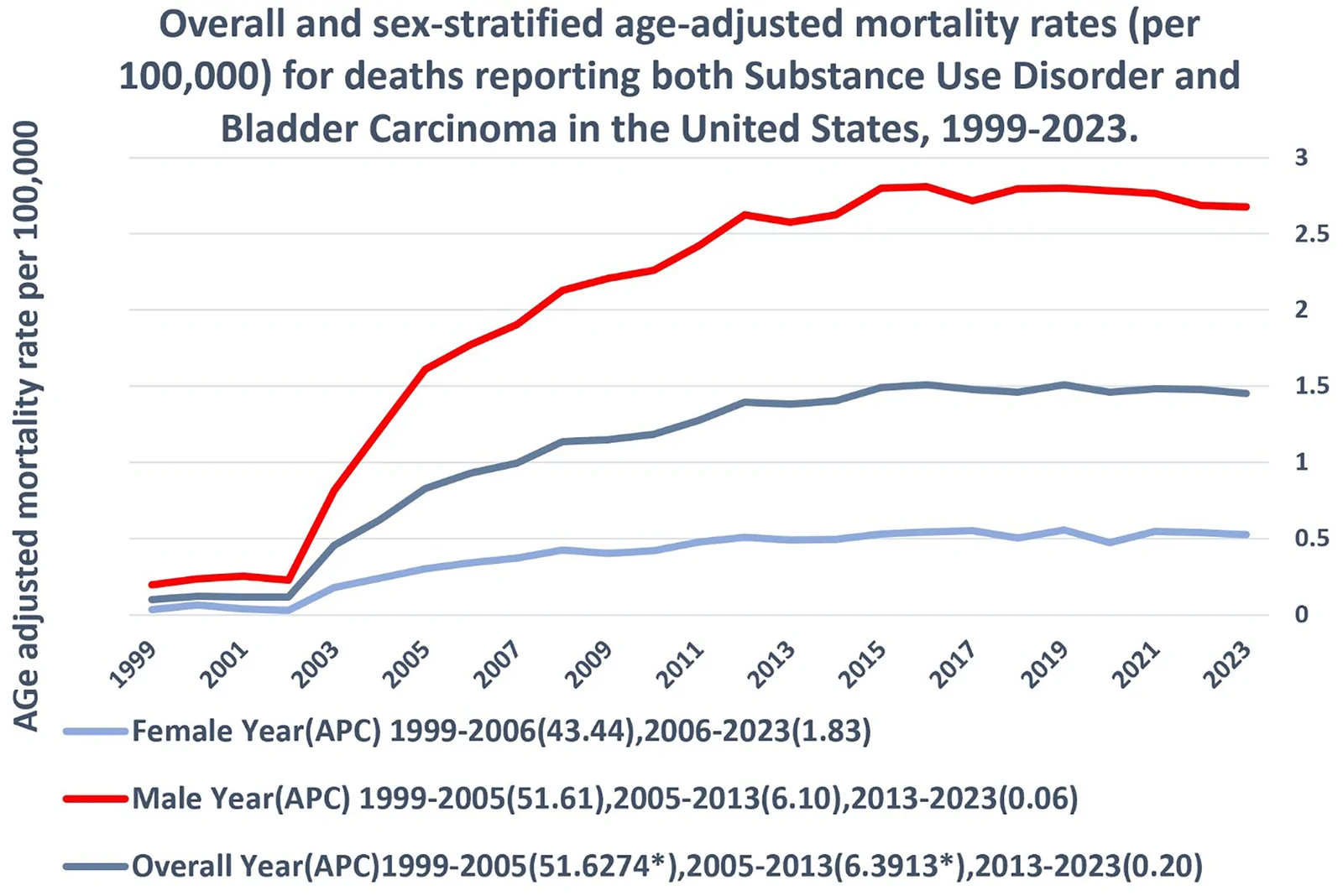
Overall and sex stratified age-adjusted mortality rates (per 100,000) for bladder cancer and substance use disorder related mortality among U.S. adults aged ≥25 years, 1999 to 2023.

**Table 2:** Annual percent change (APC) and average annual Percent change (AAPC) in age-adjusted mortality rates for deaths co-occurring with bladder cancer and substance use disorders among U.S. adults, 1999–2023.

Segment	APC (95% CI)	p-value	AAPC (95% CI)	p-value
Overall				
1999–2005	51.63* (38.21–66.35)	<0.000001		
2005–2013	6.39* (3.26–9.62)	0.000418		
2013–2023	0.20 (–1.18–1.61)	0.761511		
1999–2023			13.38* (10.69–16.14)	<0.000001
Sex				
Female				
1999–2006	43.45* (28.77–59.80)	0.000001		
2006–2023	1.83* (0.74–2.93)	0.002139		
1999–2023			12.54* (9.16–16.01)	<0.000001
Male				
1999–2005	51.61* (38.88–65.51)	<0.000001		
2005–2013	6.10* (3.18–9.11)	0.000337		
2013–2023	0.06 (–1.23–1.36)	0.924536		
1999–2023			13.20* (10.66–15.80)	<0.000001
Race/Ethnicity				
White				
1999–2005	50.70* (37.49–65.17)	<0.000001		
2005–2013	7.03* (3.90–10.25)	0.000157		
2013–2023	0.54 (–0.86–1.95)	0.429855		
1999–2023			13.59* (10.91–16.33)	<0.000001
Black or African American				
2003–2010	12.92* (6.70–19.50)	0.000331		
2010–2023	1.90* (0.57–3.25)	0.007789		
2003–2023			5.63* (3.54–7.76)	<0.000001
Hispanic or Latino				
2004–2006	39.79* (15.20–69.64)	0.003528		
2006–2013	–2.43* (–4.68 to –0.13)	0.040678		
2013–2016	14.04* (1.16–28.57)	0.035018		
2016–2023	–0.21 (–1.41–1.01)	0.701609		
2004–2023			4.72* (2.09–7.43)	0.000390
Age group				
45–54 years				
2003–2005	36.33* (0.08–85.70)	0.049505		
2005–2012	8.12* (4.42–11.96)	0.000324		
2012–2023	–3.82* (–5.12 to –2.49)	0.000036		
2003–2023			3.76* (0.60–7.02)	0.019214
55–64 years				
1999–2005	46.21* (30.01–64.43)	0.000003		
2005–2018	3.67* (2.05–5.31)	0.000156		
2018–2023	–4.71 (–9.63–0.47)	0.071222		
1999–2023			11.01* (7.71–14.41)	<0.000001
65–74 years				
1999–2005	45.02* (31.09–60.43)	0.000001		
2005–2012	6.24* (1.40–11.32)	0.014035		
2012–2023	–0.52 (–1.91–0.89)	0.443886		
1999–2023			11.43* (8.43–14.51)	<0.000001
75–84 years				
1999–2005	53.06* (38.65–68.97)	<0.000001		
2005–2012	7.42* (3.05–11.98)	0.002047		
2012–2023	0.12 (–1.23–1.49)	0.851479		
1999–2023			13.64* (10.70–16.66)	<0.000001
85+ years				
1999–2005	55.39* (38.47–74.39)	<0.000001		
2005–2014	8.02* (5.16–10.95)	0.000012		
2014–2023	2.87* (1.23–4.53)	0.001729		
1999–2023			16.15* (12.84–19.56)	<0.000001
Urbanization (1999–2020)				
Metropolitan				
1999–2006	43.90* (32.54–56.22)	<0.000001		
2006–2020	2.38* (1.26–3.52)	0.000307		
1999–2020			14.68* (11.70–17.74)	<0.000001
Nonmetropolitan				
1999–2001	–2.55 (–49.57–88.31)	0.932855		
2001–2004	76.09* (9.71–182.63)	0.023330		
2004–2012	10.57* (7.34–13.89)	0.000012		
2012–2020	1.30 (–0.54–3.18)	0.148724		
1999–2020			12.93* (3.94–22.70)	0.004080
Census region				
Northeast				
1999–2006	54.81* (37.62–74.14)	<0.000001		
2006–2023	0.08 (–0.98–1.15)	0.876744		
1999–2023			13.66* (9.97–17.47)	<0.000001
Midwest				
1999–2001	–2.85 (–38.72–54.02)	0.892711		
2001–2004	76.82* (22.02–156.24)	0.006122		
2004–2008	28.20* (18.64–38.54)	<0.000001		
2008–2013	6.74* (3.18–10.41)	0.001402		
2013–2023	–0.76* (–1.50 to –0.02)	0.044843		
1999–2023			12.82* (6.76–19.23)	0.000019
South				
2001–2005	53.98* (28.26–84.84)	0.000101		
2005–2023	3.63* (2.88–4.38)	<0.000001		
2001–2023			11.36* (7.91–14.92)	<0.000001
West				
1999–2004	50.16* (30.17–73.21)	0.000014		
2004–2013	5.91* (2.60–9.33)	0.001380		
2013–2023	–0.02 (–1.80–1.78)	0.978093		
1999–2023			11.20* (7.85–14.65)	<0.000001

### 3.2 Sex-specific trends

Among females, mortality increased significantly over the study period (AAPC, 12.54%; 95% CI, 9.16–16.01; p<0.0001). From 1999 to 2006, there was a sharp rise in mortality trend (APC = 43.45%; 95% CI, 28.77–59.80; p = 0.000001), followed by a slower but sustained increase thereafter. After rising from 0.0310 per 100,000 in 1999 to a peak of 0.5574 per 100,000 in 2019, the female age-adjusted mortality rate fell with a modest decline thereafter accounting for 12,959 fatalities (20.5%). Among males, mortality increased significantly over the study period (AAPC 13.20% 95% CI, 10.66–15.80; p < 0.0001) and mortality trends among males demonstrated a rapid increase between 1999 and 2005, followed by slower increases and eventual stabilization after 2013. After rising from 0.1942 per 100,000 in 1999 to a peak of 2.8085 per 100,000 in 2016, the male age-adjusted mortality rate fell steadily thereafter accounting for 50,299 fatalities (79.5%) (**Figure 1**, **Tables 1**, **2**, **Supplementary Table S1**).

### 3.3 Race-specific trends

Mortality patterns varied descriptively across racial and ethnic groups. White individuals accounted for the largest mortality burden (57,878 deaths; 91.5%) and mortality increased significantly over the study period (AAPC, 13.59%; 95% CI, 10.91–16.33; p < 0.0001). Trends among white individuals demonstrated a rapid increase between 1999 and 2005, followed by slower increases between 2005 and 2013 and eventual stabilization after 2013. Among Black or African American people, mortality increased significantly over the study period (AAPC, 5.63%; 95% CI, 3.54–7.76; p < 0.0001), with a marked increase between 2003 and 2010 followed by a slower but continued increase from 2010 to 2023. The AAMR rate rose to 0.9116 from 0.2413 per 100,000 between 2003 and 2023 accounting for 3,186 fatalities (5.0%). Hispanic or Latino individuals accounted for 1,364 deaths (2.2%) and mortality increased significantly over the study period (AAPC,4.72%; 95% CI, 2.09–7.43; p = 0.00039). Trends demonstrated a sharp increase between 2004 and 2006, followed by a modest decline from 2006 to 2013, a subsequent increase between 2013 and 2016, and eventual stabilization after 2016. The AAMR peaked in 2017 with a value of 0.4439 per 100,000.

American Indian or Alaska Native individuals accounted for 203 fatalities (0.3%). Although there was not enough data to estimate joinpoints, the observed rates peaked in 2017 with a value of 1.3522 per 100,000. The NH Asian or Pacific Islander category accounted for 255 deaths (0.4%); for descriptive purposes, this category comprised 140 deaths among Asian individuals (0.2%) and 115 among Native Hawaiian or Pacific Islander individuals (0.2%). These groupings had low rates typically less than 0.3 per 100,000 and no joinpoint patterns were statistically significant. Other or undefined racial/ethnic groupings accounted for the remaining 372 deaths (0.6%) (**Figure 2**, **Table 2**, **Supplementary Table S2**).

**Figure 2:**
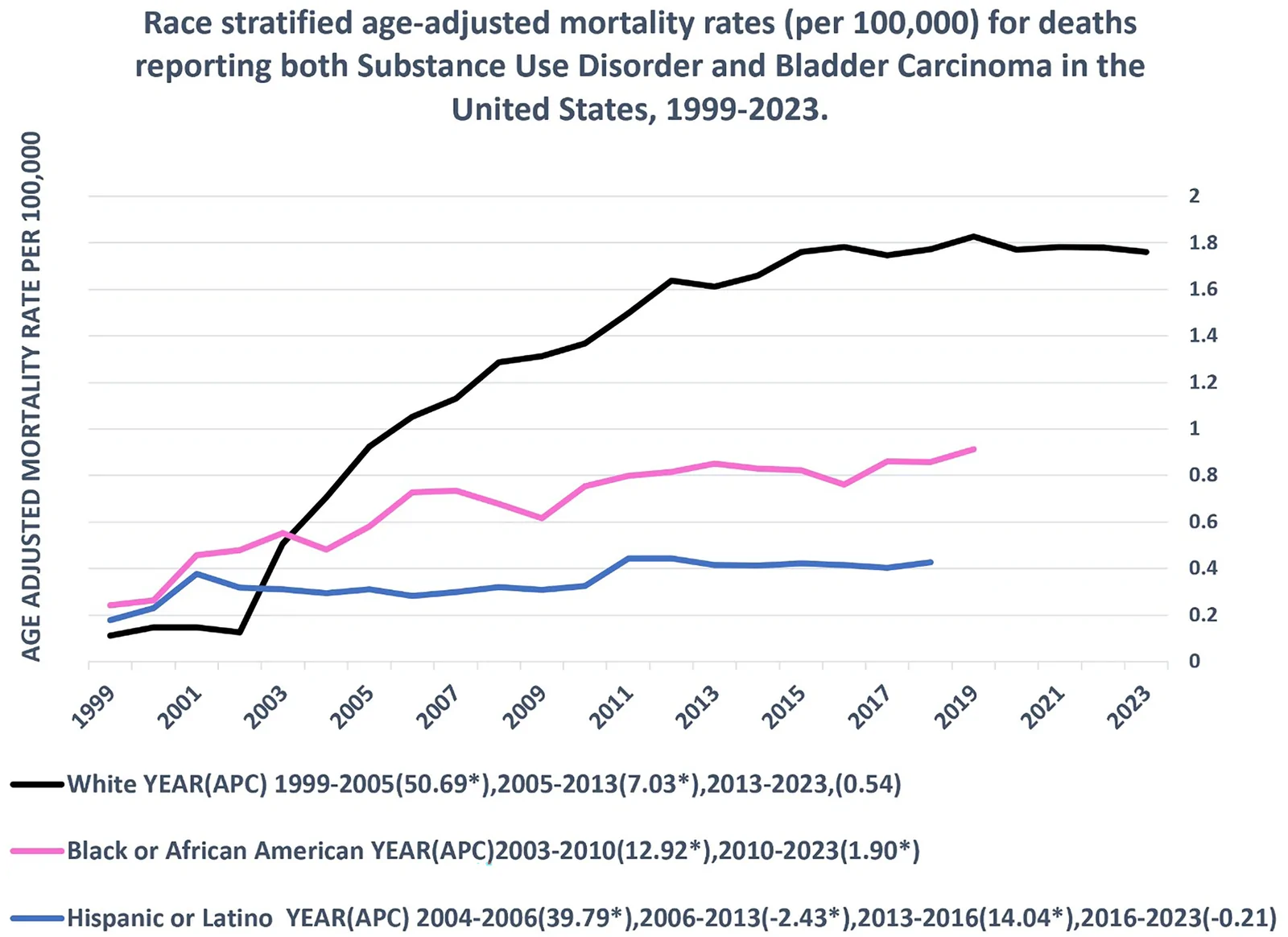
Race stratified age-adjusted mortality rates (per 100,000) for bladder cancer and substance use disorder related mortality among U.S. adults aged ≥25 years, 1999 to 2023.

### 3.4 Age group-specific trends

The analysis revealed a distinct age gradient in the death burden and trends. Mortality rates increased across all age groups, with higher mortality rates observed among older age groups. The lowest overall increase was seen in adults aged 45–54 (AAPC = 3.76%; 95% CI, 0.60–7.02; p = 0.0192), whose rates peaked in 2011 at 1.2279 per 100,000 before sharply falling after 2012. In contrast, the death rate for those aged 55–64 years increased significantly overall (AAPC = 11.01%; 95% CI, 7.71–14.41; p < 0.0001), peaking in 2016 with a value of 5.3587 from 0.4936 per 100,000.

Mortality also increased substantially among older age groups. The total AAPCs for the 65–74, 75–84, and ≥85 age categories were 11.43%, 13.64%, and 16.15%, respectively (all p < 0.0001). Over the course of the research, mortality rates rose steadily in all of these groups, with the population over 85 years old bearing the greatest burden, going from 0.1256 to 3.3767 per 100,000 (**Figure 3**, **Supplementary Table S3**).

**Figure 3:**
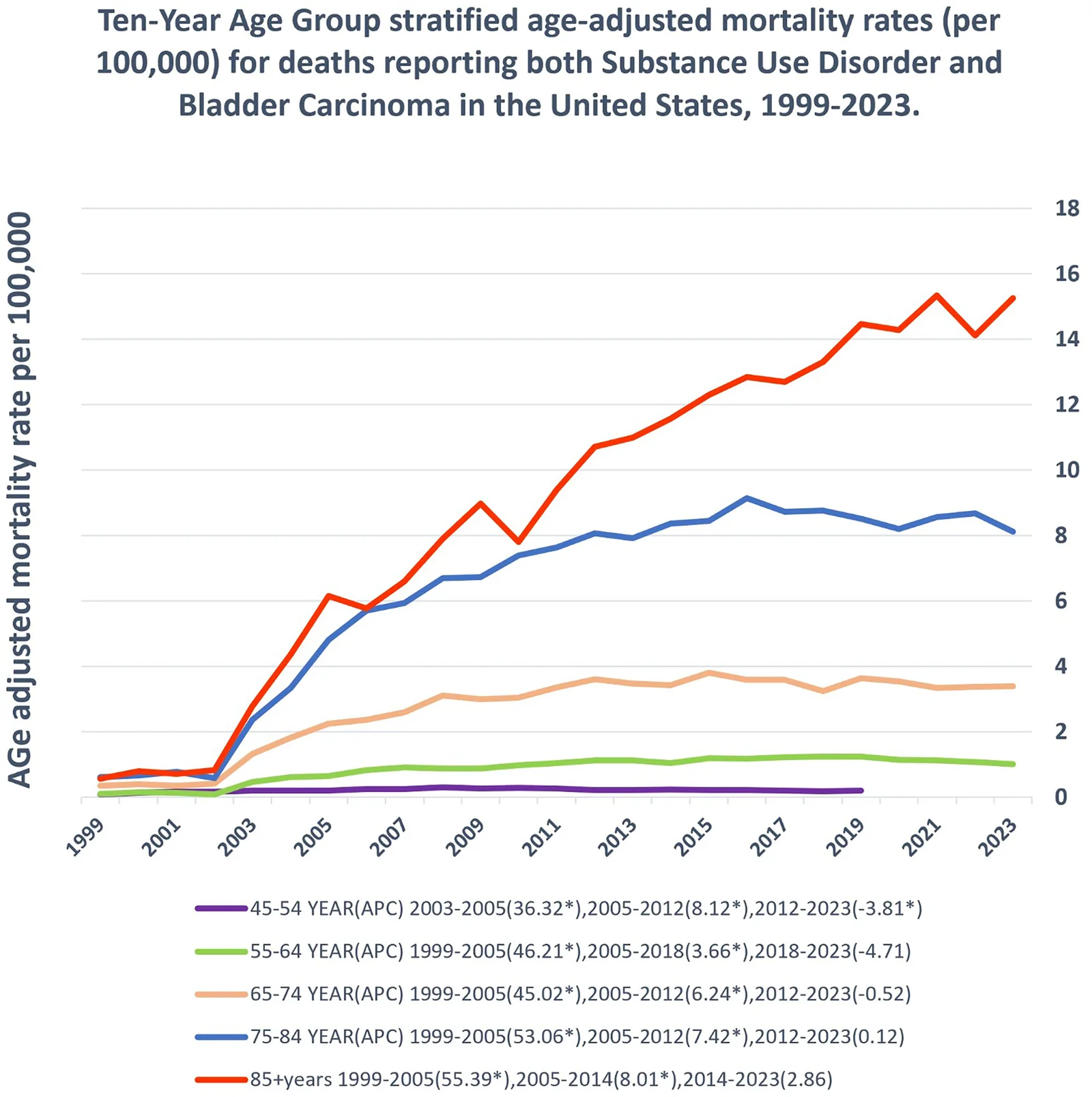
Age stratified age-adjusted mortality rates per 100,000 for bladder cancer and substance use disorder related mortality in United States adults aged ≥25 years, 1999-2023.

### 3.5 Urbanization-specific trends

Throughout the research period, mortality rates rose substantially in both metropolitan and nonmetropolitan areas. Mortality rates increased from 0.0813 to 1.4889 per 100,000 between 1999 and 2019, with an AAPC of 14.68% (95% CI, 11.70–17.74; p < 0.0001) in metropolitan regions. This surge was marked by a rapid early rise that was followed by slower but steady increases in subsequent years. Over the course of the research, mortality rates were higher in nonmetropolitan than metropolitan regions during the study period, rising from 0.2116 to a peak of 1.7347 per 100,000 between 1999 and 2015 (AAPC 12.93%; 95% CI, 3.94–22.70; p = 0.00408) (**Figure 4**, **Table 2**, **Supplementary Table S4**).

**Figure 4:**
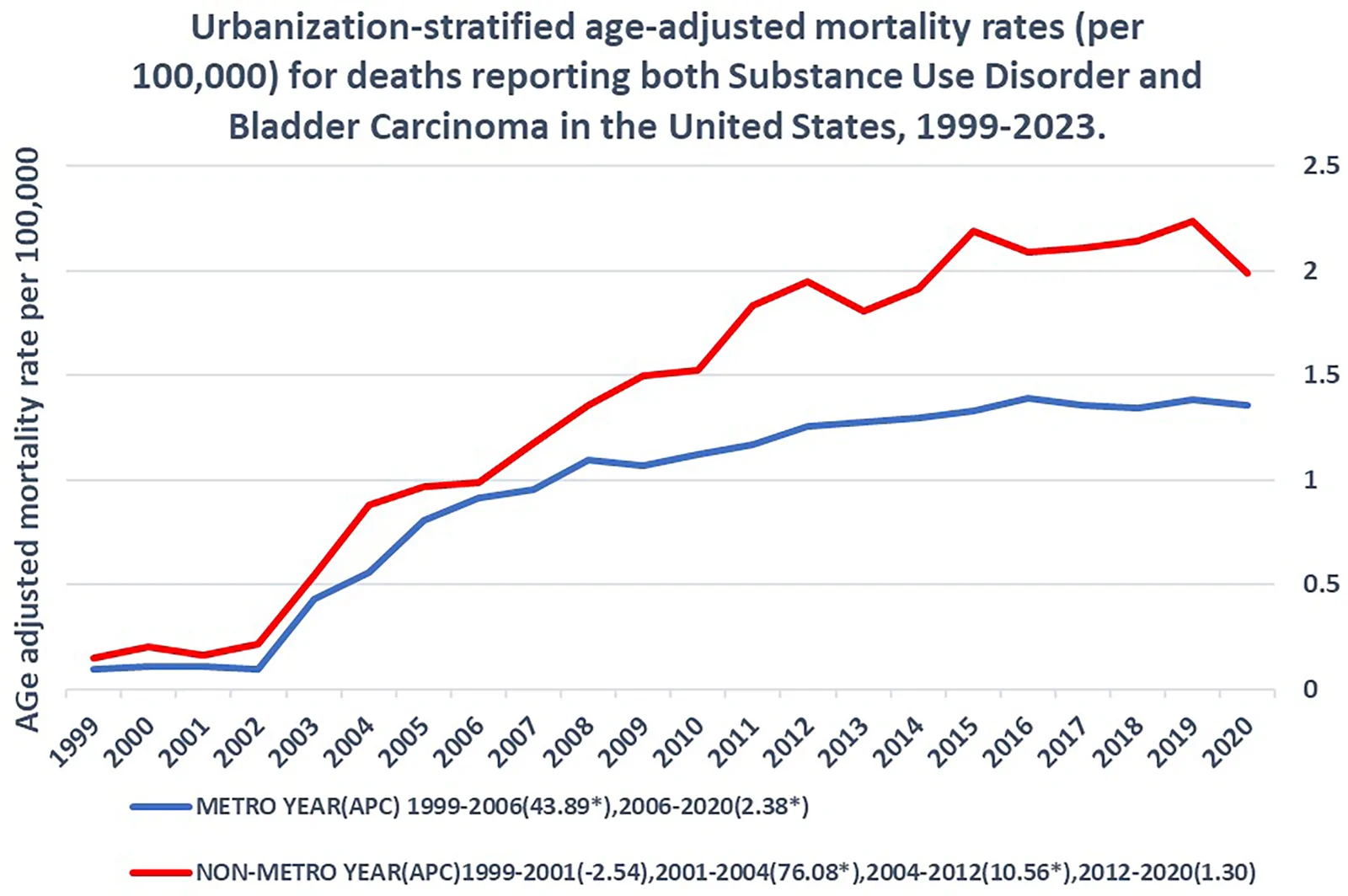
Urbanization stratified age-adjusted mortality rates per 100,000 for bladder cancer and substance use disorder related mortality in United States adults aged ≥25 years, 1999-2020.

### 3.6 Census region-specific trends

Mortality increased over the study period across all four U.S. Census regions, although the magnitude and temporal patterns varied. The Northeast had an AAPC of 13.66% (95% CI, 9.97–17.47; p < 0.0001), followed by the Midwest (AAPC, 12.82%; 95% CI, 6.76–19.23; p = 0.000019), South (AAPC, 11.36%; 95% CI, 7.91–14.92; p < 0.0001), and West (AAPC, 11.20%; 95% CI, 7.85–14.65; p < 0.0001) (**Table 2**). Observed mortality rates peaked at 1.3104 per 100,000 in the Northeast, 1.9529 per 100,000 in the Midwest, 1.3289 per 100,000 in the South, and 1.5332 per 100,000 in the West.

From 1999 to 2006, the Northeast had a sharp early spike (APC = 54.81%; p < 0.0001), which was followed by a protracted plateau until 2023. In a similar vein, the South showed a consistent increasing tendency starting in 2001, but the West had a quick initial increase, slower development, and a non-significant trend in its most recent phase (2013–2023; APC = –0.02%; p = 0.9781). The country’s overall regional death burden rose, with the Northeast and Midwest showing comparatively sharper rises (**Figure 5**, **Table 2**, **Supplementary Table S5**).

**Figure 5:**
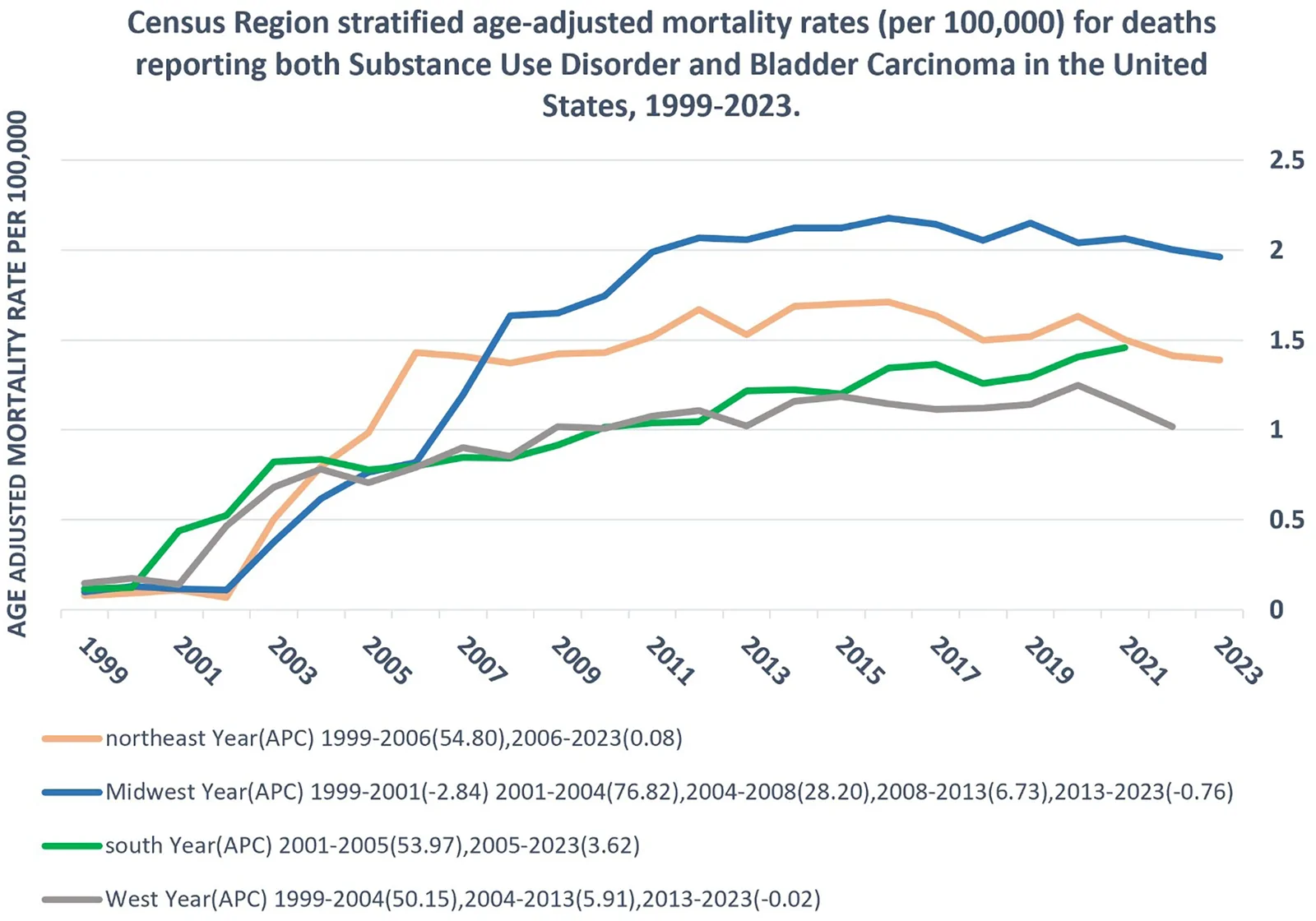
Census region stratified age-adjusted mortality rates per 100,000 for bladder cancer and substance use disorder related mortality in United States adults aged ≥25 years, 1999-2023.

**Figure 6:**
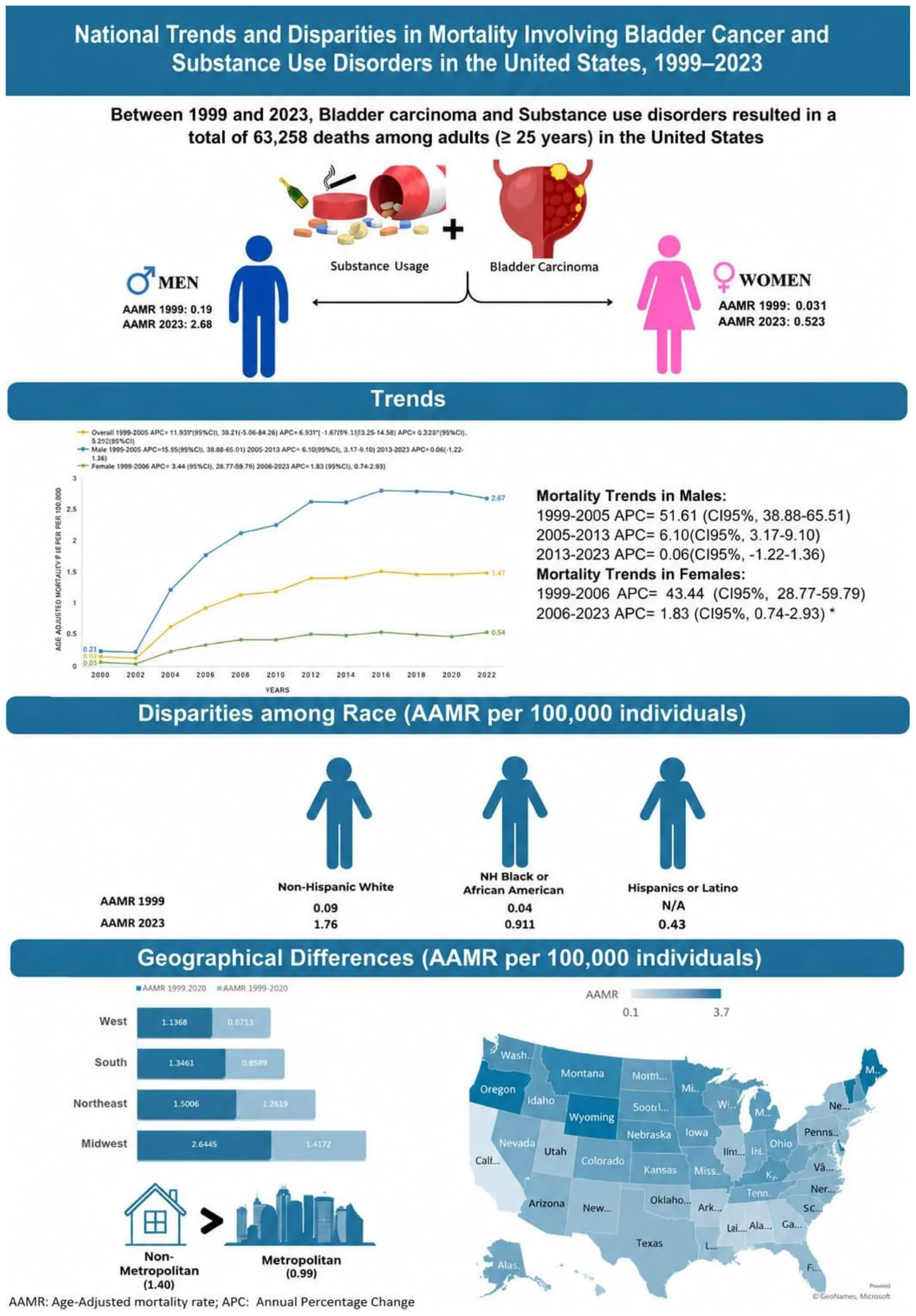
Graphical abstract summarizing national trends and disparities in mortality involving bladder cancer and substance use disorders among U.S. adults aged ≥25 years from 1999 to 2023.

### 3.7 States-specific trends

Between 1999 and 2023, SUDs and bladder cancer claimed 63,250 lives in the 50 states and the District of Columbia (51,057 fatalities from 1999 to 2020 and 12,193 from 2021 to 2023). The summed state-level total is slightly lower than the national total of 63,258 deaths because CDC WONDER suppresses state-level counts fewer than 10 to protect confidentiality. Texas (4,582 fatalities, 7.24% of total), New York (4,439 deaths, 7.02%), Florida (4,292 deaths, 6.79%), Ohio (3,619 deaths, 5.72%), and Michigan (3,404 deaths, 5.38%) recorded the highest numbers of deaths. Illinois (2,041), Wisconsin (2,386), Washington (2,709), and Oregon (2,215), all reported more than 2,000 deaths and Pennsylvania (3,132) was the only other state with more than 3,000 fatalities The states with the fewest deaths were North Dakota (335), Mississippi (118), Alaska (113), and the District of Columbia (59). Temporal trends revealed that 81% of fatalities took place between 1999 and 2020, with the latter three years (2021–2023) accounting for the remaining 19% (**Supplementary Figure S1**, **Supplementary Tables S6**, **S7**).

### 3.8 Place of death-specific trends

Among place of death, home was the most common location (47.0%), followed by inpatient medical facilities (22.6%), nursing homes/long-term care facilities (13.5%), and hospice facilities (11.4%). The proportion of deaths occurring at home increased over time, whereas inpatient facility deaths declined modestly between 1999–2020 and 2021–2023 (**Supplementary Tables S8****).**

## 4 Discussion

In this retrospective analysis, we identified 63,258 deaths involving co-occurring bladder cancer and substance use disorders (SUDs) in the United States. Mortality increased substantially over the study period and varied markedly by age, sex, race/ethnicity, and geographic region. The estimated prevalence of SUDs in US adult cancer survivors is approximately 3.8% but varies by cancer type and is estimated to be 1.93% for bladder cancer (7). Concomitant SUDs may complicate cancer management, owing to challenges related to treatment adherence, pain management, and healthcare access (13, 14). Collectively, these findings suggest that patients with coexisting bladder cancer and SUD represent a particularly vulnerable population in whom barriers to cancer treatment and addiction care may adversely affect outcomes.

Overall, coexisting bladder cancer- and SUD-related mortality increased over time (AAPC 13.4%; 95% CI: 10.7, 16.1), broadly in parallel with national increases in substance-related mortality. For context, national substance-related mortality increased from 2.22 to 24.75 per 100,000 between 1999 and 2023, with an AAPC of 10.17% (15). Whereas bladder cancer mortality has steadily declined at an average rate of 0.4% per year from 1999 to 2020 (8). Changes in ICD-10 coding may also have influenced these trends, which should be considered when interpreting temporal trends. Since 2020, the single code for synthetic narcotics has been replaced by three individual codes for fentanyl, tramadol, and others. This may have improved the identification of fentanyl-related deaths (16). The Centers for Disease Control and Prevention (CDC) has divided the opioid overdose epidemic into three surges: the first phase of prescription opioids (1999), followed by deaths from heroin use (2010), and then the synthetic opioids-related deaths (2013), which have declined in 2023 (17). Because all substance use disorders (ICD-10 F10–F19) were analyzed together, the present study cannot determine which substances drove these deaths. Thus, these temporal patterns should be interpreted with caution and not taken as conclusive evidence that any specific substance caused the observed increase. Although the timing of these changes broadly coincides with the successive phases of the opioid epidemic described by the CDC, our findings do not permit attribution of these trends to any specific substance. The observed correspondence should therefore be interpreted as a possible explanation rather than evidence of causality. These findings underscore the need for ongoing monitoring and better reporting of substance-related deaths so we can more accurately understand mortality patterns in patients with bladder cancer.

Joinpoint regression also showed differences across racial and ethnic groups in coexisting bladder cancer- and SUD-related mortality. The observed differences between White and Black populations may partially reflect the ‘bladder cancer paradox’ described in prior literature. Black women have a 36% lower incidence of bladder cancer as compared to White women. However, Schafer et al. (2023) suggest that they are almost equal, attributing the same differences in access to healthcare, later stage diagnosis, and lower survival among Black patients (18). National survey data from 2015–2019 indicate that Hispanic and Black populations have lower prevalence of SUD than White populations, which may partly contribute to the observed differences in their lower AAPCs. The ASPE report also identified a 38 percent increase in the rate of opioid-related death for Black people from 2018 to 2019 (19). There are also sex differences in mortality due to bladder cancer and SUDs. The higher mortality in men is probably a consequence of a longer lifetime exposure to smoking and occupational carcinogens (20). Women are most likely to experience diagnostic delays because early bladder cancer symptoms are misdiagnosed and treated for other conditions, most commonly urinary tract infections. Women are more likely to be diagnosed at a later stage, associated with worse outcomes and higher mortality (21). The disparity is likely due to a combination of structural barriers, differential exposure to risk factors, including smoking and occupational carcinogens, and diagnostic delays. Addressing disparities in timely diagnosis and equitable access to both oncologic and addiction care may address these differences in mortality.

We also saw regional differences in mortality, probably reflecting regional differences in rates of substance-related deaths, access to healthcare, and population density. 2022 CDC data indicates West Virginia had the highest AAMR for drug overdose. Ohio and Michigan from the Midwest region have the highest overdose rates (22). On the other hand, New York and Florida have large populations, which leads to a larger number of deaths despite having the same overdose rates. Such findings could reflect differences in rates of substance use disorders, access to health care, and population density among regions. Bladder cancer has been linked to certain occupations, especially those involving exposure to aromatic amines (in dyes, paint, and rubber). Industries involving aromatic amine exposure are concentrated in states such as Ohio, Michigan, Pennsylvania, and New Jersey (23). Exploring these regional industrial trends may help to identify potential contributory factors. Cancer death rates are highly dependent on healthcare access. Geographic variation in mortality may also be influenced by variation in Medicaid expansion policies (24).According to the American Urological Association (AUA), the number of urologists practicing in rural areas is below what would be expected based on the population size of those areas (25, 26). Patients in rural areas may also experience delays in diagnosis and treatment because of limited access to urologic and addiction services. Thus, increasing access to both oncologic and addiction services in underserved areas can help to reduce these observed geographic differences in mortality. Differences across age groups may reflect underlying patterns of SUDs.

The age gradient identified in our study may be related to changes in substance exposure across birth cohorts and the increasing incidence of bladder cancer with age. The 45 to 54-year age group might have different patterns of substance exposure, reflecting cohort-based prescribing patterns as described by the phases of the opioid overdose epidemic (20). Moreover, middle-aged individuals face multiple mortality risks simultaneously, like cardiovascular diseases, stress, accidents, or any malignancy. According to the National Cancer Institute report, the incidence of bladder cancer increases with age (27). These findings support age-targeted prevention strategies and closer clinical follow-up for patients with overlapping risks related to bladder cancer and substance use.

### 4.1 Limitations

This study has some limitations. Firstly, the death certificate data is often imprecise. A recent study estimated that 20–30% of drug-related deaths are registered in the CDC database with incomplete cause-of-death information (28). Consequently, Coding of substance use on death certificates likely changed over time which may also capture incomplete information on polysubstance usage. Furthermore, individual-level causal relationships may not reflect the population-level associations underlying these findings, a phenomenon known as the ecological fallacy. Comparisons across demographic and geographic subgroups were descriptive in nature, and no formal statistical tests for subgroup differences or interactions were performed. Additionally, death certificate data may incompletely capture polysubstance use patterns and other co-occurring clinical conditions, which may underestimate the complexity of modern multi-substance fatal overdoses and make it difficult to identify specific trends regarding rare disorders.

### 4.2 Conclusion

Mortality involving bladder cancer and coexisting substance use disorders increased significantly in the United States from 1999 to 2023, with notable variations by sex, race/ethnicity, age, and geography. Unexpectedly high rates of mortality were observed among older adults and persons living in nonmetropolitan areas. These findings are consistent with national trends in SUD–related mortality and may reflect ongoing differences in availability of oncology and addiction treatment services. Integrated clinical and public health approaches to care for both cancer and substance use may help to mitigate this growing mortality burden.

## Data Availability

Publicly available datasets were analyzed in this study. This data can be found here: https://wonder.cdc.gov/.
