# Supplementary material for: National trends and disparities in mortality involving bladder cancer and substance use disorders in the United States, 1999–2023
Source: Front Oncol. 2026 Sep 29;16:1883338. doi: 10.3389/fonc.2026.1883338 (PMC13657952; doi:10.3389/fonc.2026.1883338)
Supplement: Supplementary file 1 [file SupplementaryFile1.docx]

**SUPPLEMENTARY MATERIAL**

**Supplemental Table 1:** Overall and sex-stratified age-adjusted mortality rates (per 100,000) for drug abuse and bladder cancer in the United States, 1999–2023.

**Supplemental Table 2:** Race- and ethnicity-stratified age-adjusted mortality rates (per 100,000) for drug abuse and bladder cancer in the United States, 1999–2023.

**Supplemental Table 3:** Age group–stratified age-adjusted mortality rates (per 100,000) for drug abuse and bladder cancer in the United States, 1999–2023.

**Supplemental Table 4:** Urbanization-stratified age-adjusted mortality rates (per 100,000) for drug abuse and bladder cancer in the United States, 1999–2020.

**Supplemental Table 5:** Census region–stratified mortality rates (per 100,000) for drug abuse and bladder cancer in the United States, 1999-2023.

**Supplemental Table 6:** State-level death counts (per 100,000) for drug abuse and bladder cancer, United States, 1999–2023.

**Supplemental Table 7:** State-level death counts for drug abuse and bladder cancer, United States, 1999–2023.

**Supplemental Table 8:** Percentage distribution of place of death for drug abuse and bladder cancer fatalities, United States, 1999–2023.

**Supplementary Figure 1:** State stratified age-adjusted mortality rates per 100,000 for drug abuse and bladder cancer fatalities related mortality in United States, 1999-2023.

**Supplementary Figure 2:** Percentage distribution of place of death in the United States (1999–2023), for drug abuse and bladder cancer fatalities related mortality highlighted

**Supplemental Table 1:** Overall and sex-stratified age-adjusted mortality rates (per 100,000) for drug abuse and bladder cancer in the United States, 1999–2023.

| **Year** | **Overall AAMR (95% CI)** | **Women AAMR (95% CI)** | **Men AAMR (95% CI)** |
| --- | --- | --- | --- |
| 1999 | 0.1001 (0.0854–0.1149) | 0.0310 (0.0217–0.0429) | 0.1942 (0.1611–0.2274) |
| 2000 | 0.1221 (0.1059–0.1382) | 0.0619 (0.0455–0.0823) | 0.2366 (0.2006–0.2726) |
| 2001 | 0.1164 (0.1010–0.1318) | 0.0382 (0.0277–0.0515) | 0.2539 (0.2163–0.2915) |
| 2002 | 0.1151 (0.0993–0.1310) | 0.0289 (0.0199–0.0406) | 0.2242 (0.1896–0.2589) |
| 2003 | 0.4546 (0.4237–0.4855) | 0.1786 (0.1528–0.2043) | 0.8151 (0.7508–0.8793) |
| 2004 | 0.6200 (0.5848–0.6552) | 0.2406 (0.2107–0.2706) | 1.2128 (1.1340–1.2916) |
| 2005 | 0.8265 (0.7859–0.8670) | 0.3017 (0.2692–0.3342) | 1.6084 (1.5186–1.6983) |
| 2006 | 0.9291 (0.8861–0.9720) | 0.3399 (0.3052–0.3746) | 1.7711 (1.6782–1.8639) |
| 2007 | 0.9960 (0.9522–1.0398) | 0.3727 (0.3371–0.4083) | 1.9064 (1.8110–2.0018) |
| 2008 | 1.1338 (1.0873–1.1802) | 0.4231 (0.3854–0.4609) | 2.1272 (2.0280–2.2264) |
| 2009 | 1.1499 (1.1036–1.1963) | 0.4024 (0.3661–0.4388) | 2.2057 (2.1053–2.3061) |
| 2010 | 1.1832 (1.1366–1.2297) | 0.4211 (0.3837–0.4585) | 2.2589 (2.1588–2.3589) |
| 2011 | 1.2763 (1.2286–1.3240) | 0.4763 (0.4376–0.5151) | 2.4249 (2.3224–2.5274) |
| 2012 | 1.3969 (1.3474–1.4464) | 0.5078 (0.4677–0.5478) | 2.6255 (2.5205–2.7304) |
| 2013 | 1.3800 (1.3313–1.4287) | 0.4905 (0.4518–0.5291) | 2.5753 (2.4729–2.6777) |
| 2014 | 1.4054 (1.3567–1.4540) | 0.4944 (0.4567–0.5321) | 2.6230 (2.5206–2.7253) |
| 2015 | 1.4900 (1.4406–1.5394) | 0.5302 (0.4914–0.5690) | 2.7990 (2.6944–2.9036) |
| 2016 | 1.5092 (1.4601–1.5583) | 0.5408 (0.5016–0.5800) | 2.8085 (2.7054–2.9115) |
| 2017 | 1.4791 (1.4313–1.5269) | 0.5497 (0.5111–0.5884) | 2.7188 (2.6191–2.8185) |
| 2018 | 1.4596 (1.4127–1.5064) | 0.5033 (0.4662–0.5405) | 2.7943 (2.6939–2.8948) |
| 2019 | 1.5084 (1.4615–1.5552) | 0.5574 (0.5191–0.5958) | 2.7991 (2.7009–2.8973) |
| 2020 | 1.4591 (1.4135–1.5047) | 0.4738 (0.4392–0.5084) | 2.7826 (2.6857–2.8794) |
| 2021 | 1.4835 (1.4367–1.5302) | 0.5476 (0.5093–0.5859) | 2.7641 (2.6655–2.8627) |
| 2022 | 1.4788 (1.4330–1.5245) | 0.5401 (0.5037–0.5764) | 2.6877 (2.5933–2.7820) |
| 2023 | 1.4524 (1.4076–1.4973) | 0.5229 (0.4870–0.5588) | 2.6796 (2.5859–2.7733) |

**Supplemental Table 2:** Race- and ethnicity-stratified age-adjusted mortality rates (per 100,000) for drug abuse and bladder cancer in the United States, 1999–2023.

| **Year** | **American Indian or Alaska Native (95% CI)** | **Asian or Pacific Islander (95% CI)** | **Black or African American (95% CI)** | **White (95% CI)** | **Asian (95% CI)** | **Hispanic or Latino (95% CI)** |
| --- | --- | --- | --- | --- | --- | --- |
| 1999 | – | – | – | 0.1103 (0.0933–0.1273) | – | – |
| 2000 | – | – | – | 0.1458 (0.1259–0.1657) | – | – |
| 2001 | – | – | – | 0.1470 (0.1271–0.1670) | – | – |
| 2002 | – | – | – | 0.1245 (0.1066–0.1423) | – | – |
| 2003 | – | – | 0.2413 (0.1690–0.3340) | 0.5072 (0.4714–0.5431) | – | – |
| 2004 | – | – | 0.2624 (0.1883–0.3560) | 0.7060 (0.6645–0.7474) | – | 0.1785 (0.1075–0.2788) |
| 2005 | 0.4242 (0.1845–0.8760)* | – | 0.4582 (0.3585–0.5770) | 0.9242 (0.8771–0.9713) | – | 0.2287 (0.1450–0.3431) |
| 2006 | – | – | 0.4783 (0.3762–0.5995) | 1.0526 (1.0022–1.1031) | – | 0.3770 (0.2706–0.5115) |
| 2007 | – | 0.0603 (0.0415–0.0842) | 0.5510 (0.4447–0.6750) | 1.1305 (1.0788–1.1822) | – | 0.3183 (0.2253–0.4369) |
| 2008 | 0.8734 (0.5008–1.4050) | 0.1310 (0.0743–0.2133) | 0.4803 (0.3809–0.5978) | 1.2868 (1.2321–1.3416) | – | 0.3109 (0.2241–0.4202) |
| 2009 | – | – | 0.5815 (0.4652–0.6978) | 1.3132 (1.2584–1.3681) | – | 0.2940 (0.2080–0.4035) |
| 2010 | 0.4433 (0.2209–0.7932) | 0.1099 (0.0610–0.1805) | 0.7267 (0.6005–0.8529) | 1.3682 (1.3120–1.4244) | – | 0.3094 (0.2220–0.4197) |
| 2011 | 0.4317 (0.2071–0.7940) | 0.0980 (0.0541–0.1615) | 0.7341 (0.6105–0.8577) | 1.4982 (1.4402–1.5563) | – | 0.2817 (0.1994–0.3867) |
| 2012 | – | 0.0719 (0.0380–0.1230) | 0.6778 (0.5611–0.7945) | 1.6375 (1.5773–1.6977) | – | 0.2983 (0.2184–0.3978) |
| 2013 | 0.3986 (0.1991–0.7126) | 0.2695 (0.1727–0.4009) | 0.6150 (0.5068–0.7231) | 1.6124 (1.5534–1.6714) | – | 0.3207 (0.2402–0.4195) |
| 2014 | 0.4225 (0.2110–0.7560) | 0.2167 (0.1324–0.3347) | 0.7544 (0.6357–0.8732) | 1.6586 (1.5988–1.7184) | – | 0.3083 (0.2296–0.4054) |
| 2015 | 0.6918 (0.3990–1.1140) | 0.1750 (0.1020–0.2803) | 0.7995 (0.6795–0.9194) | 1.7608 (1.6997–1.8218) | – | 0.3238 (0.2465–0.4177) |
| 2016 | 0.4198 (0.2169–0.7334) | 0.2212 (0.1402–0.3320) | 0.8154 (0.6978–0.9331) | 1.7818 (1.7208–1.8428) | – | 0.4422 (0.3522–0.5481) |
| 2017 | 1.3522 (0.8141–2.1116) | 0.2574 (0.1711–0.3720) | 0.8508 (0.7301–0.9714) | 1.7467 (1.6873–1.8062) | – | 0.4439 (0.3555–0.5475) |
| 2018 | – | 0.0853 (0.0475–0.1412) | 0.8287 (0.7135–0.9438) | 1.7722 (1.7118–1.8325) | 0.0542 (0.0306–0.1833) | 0.4142 (0.3303–0.5128) |
| 2019 | 0.5756 (0.3287–0.9402) | 0.0585 (0.0334–0.1795) | 0.8233 (0.7092–0.9374) | 1.8280 (1.7684–1.8876) | 0.0475 (0.0269–0.1606) | 0.4128 (0.3291–0.4964) |
| 2020 | 0.5748 (0.3401–0.9090) | 0.2283 (0.1540–0.3259) | 0.7617 (0.6529–0.8704) | 1.7709 (1.7127–1.8291) | 0.1998 (0.1305–0.2927) | 0.4224 (0.3417–0.5163) |
| 2021 | – | – | 0.8596 (0.7406–0.9785) | 1.7828 (1.7241–1.8416) | 0.1889 (0.1210–0.2811) | 0.4151 (0.3318–0.4984) |
| 2022 | 1.3424 (0.8413–2.0324) | – | 0.8581 (0.7438–0.9724) | 1.7795 (1.7211–1.8378) | 0.2474 (0.1703–0.3475) | 0.4016 (0.3232–0.4799) |
| 2023 | 0.4303 (0.2405–0.7238) | – | 0.9116 (0.7953–1.0278) | 1.7603 (1.7026–1.8181) | 0.1715 (0.1110–0.2532) | 0.4276 (0.3475–0.5076) |

**Supplemental Table 3.** Age group–stratified age-adjusted mortality rates (per 100,000) for drug abuse and bladder cancer in the United States, 1999–2023.

| **Year** | **45–54 years AAMR (95% CI)** | **55–64 years AAMR (95% CI)** | **65–74 years AAMR (95% CI)** | **75–84 years AAMR (95% CI)** | **85+ years AAMR (95% CI)** |
| --- | --- | --- | --- | --- | --- |
| 1999 | – | 0.4936 (0.3534–0.6338) | 0.4580 (0.3596–0.5564) | 0.2629 (0.1961–0.3297) | 0.1256 (0.0638–0.1874) |
| 2000 | – | 0.4786 (0.3326–0.6246) | 0.6288 (0.5173–0.7403) | 0.3459 (0.2709–0.4209) | 0.1308 (0.0640–0.1976) |
| 2001 | – | 0.4898 (0.3438–0.6358) | 0.5794 (0.4703–0.6885) | 0.3272 (0.2536–0.4008) | 0.1045 (0.0435–0.1655) |
| 2002 | – | 0.4481 (0.3066–0.5896) | 0.5864 (0.4774–0.6954) | 0.3493 (0.2732–0.4254) | 0.1640 (0.0900–0.2380) |
| 2003 | 0.3964 (0.2579–0.5349) | 1.5137 (1.2596–1.7678) | 1.4971 (1.3205–1.6737) | 0.9616 (0.8393–1.0839) | 0.3038 (0.1939–0.4137) |
| 2004 | 0.5602 (0.3967–0.7237) | 2.0735 (1.7671–2.3799) | 1.9287 (1.7300–2.1274) | 1.3408 (1.1980–1.4836) | 0.5480 (0.4054–0.6906) |
| 2005 | 0.7708 (0.5809–0.9607) | 2.9697 (2.5953–3.3441) | 2.6820 (2.4469–2.9171) | 1.7577 (1.5960–1.9194) | 0.7138 (0.5516–0.8760) |
| 2006 | 0.9095 (0.7009–1.1181) | 3.3896 (2.9835–3.7957) | 2.9792 (2.7363–3.2221) | 1.9561 (1.7870–2.1252) | 0.9459 (0.7602–1.1316) |
| 2007 | 0.8633 (0.6568–1.0698) | 3.7747 (3.3385–4.2109) | 3.3715 (3.1125–3.6305) | 2.0594 (1.8855–2.2333) | 1.1754 (0.9701–1.3807) |
| 2008 | 1.0562 (0.8300–1.2824) | 4.5521 (4.0667–5.0375) | 3.7687 (3.4986–4.0388) | 2.5216 (2.3355–2.7077) | 1.3611 (1.1407–1.5815) |
| 2009 | 1.0344 (0.8097–1.2591) | 4.5537 (4.0690–5.0384) | 4.1667 (3.8838–4.4496) | 2.6564 (2.4651–2.8477) | 1.4069 (1.1845–1.6293) |
| 2010 | 1.0237 (0.7999–1.2475) | 4.4175 (3.9372–4.8978) | 4.1598 (3.8793–4.4403) | 2.6278 (2.4372–2.8184) | 1.6045 (1.3691–1.8399) |
| 2011 | 1.2279 (0.9823–1.4735) | 4.6574 (4.1599–5.1549) | 4.4680 (4.1791–4.7569) | 2.8481 (2.6497–3.0465) | 1.7548 (1.5082–2.0014) |
| 2012 | 1.2151 (0.9700–1.4602) | 4.8196 (4.3107–5.3285) | 4.8732 (4.5727–5.1737) | 3.0976 (2.8915–3.3037) | 2.0800 (1.8155–2.3445) |
| 2013 | 1.1390 (0.8957–1.3823) | 4.7883 (4.2812–5.2954) | 4.7296 (4.4334–5.0258) | 3.1663 (2.9571–3.3755) | 2.1910 (1.9193–2.4627) |
| 2014 | 1.1531 (0.9089–1.3973) | 4.6814 (4.1779–5.1849) | 4.7700 (4.4735–5.0665) | 3.1911 (2.9812–3.4010) | 2.3454 (2.0650–2.6258) |
| 2015 | 1.1158 (0.8725–1.3591) | 5.1620 (4.6371–5.6869) | 5.0689 (4.7635–5.3743) | 3.3567 (3.1416–3.5718) | 2.5952 (2.3016–2.8888) |
| 2016 | 0.9860 (0.7484–1.2236) | 5.3587 (4.8216–5.8958) | 5.2467 (4.9382–5.5552) | 3.4761 (3.2570–3.6952) | 2.8989 (2.5914–3.2064) |
| 2017 | 0.9470 (0.7129–1.1811) | 5.2285 (4.6984–5.7586) | 5.0910 (4.7870–5.3950) | 3.4425 (3.2246–3.6604) | 2.8915 (2.5849–3.1981) |
| 2018 | 0.9284 (0.6962–1.1606) | 5.0446 (4.5226–5.5666) | 5.0024 (4.7006–5.3042) | 3.3749 (3.1595–3.5903) | 2.9457 (2.6362–3.2552) |
| 2019 | 0.9222 (0.6905–1.1539) | 5.2032 (4.6744–5.7320) | 5.1804 (4.8746–5.4862) | 3.5006 (3.2818–3.7194) | 3.1050 (2.7884–3.4216) |
| 2020 | 0.9683 (0.7335–1.2031) | 5.0601 (4.5393–5.5809) | 5.0245 (4.7209–5.3281) | 3.4043 (3.1884–3.6202) | 3.1296 (2.8129–3.4463) |
| 2021 | 0.8732 (0.6439–1.1025) | 5.2521 (4.7239–5.7803) | 5.1763 (4.8700–5.4826) | 3.4812 (3.2633–3.6991) | 3.2375 (2.9153–3.5597) |
| 2022 | 0.7680 (0.5446–0.9914) | 5.2056 (4.6800–5.7312) | 5.1218 (4.8174–5.4262) | 3.3589 (3.1434–3.5744) | 3.3767 (3.0474–3.7060) |
| 2023 | 0.6862 (0.4684–0.9040) | 5.1678 (4.6444–5.6912) | 5.1808 (4.8758–5.4858) | 3.3686 (3.1534–3.5838) | 3.3595 (3.0303–3.6887) |

**Supplemental Table 4:** Urbanization-stratified age-adjusted mortality rates (per 100,000) for drug abuse and bladder cancer in the United States, 1999–2020.

| **Year** | **Metropolitan AAMR (95% CI)** | **Nonmetropolitan AAMR (95% CI)** |
| --- | --- | --- |
| 1999 | 0.0813 (0.0640–0.0986) | 0.2116 (0.1465–0.2767) |
| 2000 | 0.1001 (0.0806–0.1196) | 0.2296 (0.1611–0.2981) |
| 2001 | 0.0937 (0.0747–0.1127) | 0.2210 (0.1538–0.2882) |
| 2002 | 0.0925 (0.0736–0.1114) | 0.2243 (0.1568–0.2918) |
| 2003 | 0.4115 (0.3794–0.4436) | 0.7223 (0.6190–0.8256) |
| 2004 | 0.5736 (0.5364–0.6108) | 0.9668 (0.8459–1.0877) |
| 2005 | 0.7717 (0.7290–0.8144) | 1.2581 (1.1157–1.4005) |
| 2006 | 0.8701 (0.8251–0.9151) | 1.3944 (1.2445–1.5443) |
| 2007 | 0.9398 (0.8931–0.9865) | 1.4344 (1.2814–1.5874) |
| 2008 | 1.0776 (1.0289–1.1263) | 1.5965 (1.4348–1.7582) |
| 2009 | 1.0968 (1.0477–1.1459) | 1.6164 (1.4528–1.7800) |
| 2010 | 1.1357 (1.0861–1.1853) | 1.6153 (1.4505–1.7801) |
| 2011 | 1.2361 (1.1857–1.2865) | 1.6469 (1.4789–1.8149) |
| 2012 | 1.3595 (1.3075–1.4115) | 1.7301 (1.5579–1.9023) |
| 2013 | 1.3400 (1.2884–1.3916) | 1.7353 (1.5628–1.9078) |
| 2014 | 1.3745 (1.3226–1.4264) | 1.6997 (1.5273–1.8721) |
| 2015 | 1.4617 (1.4087–1.5147) | 1.7347 (1.5574–1.9120) |
| 2016 | 1.4882 (1.4347–1.5417) | 1.6660 (1.4915–1.8405) |
| 2017 | 1.4541 (1.4012–1.5070) | 1.6792 (1.5029–1.8555) |
| 2018 | 1.4382 (1.3855–1.4909) | 1.5965 (1.4232–1.7698) |
| 2019 | 1.4889 (1.4355–1.5423) | 1.6230 (1.4467–1.7993) |
| 2020 | 1.4419 (1.3892–1.4946) | 1.5597 (1.3850–1.7344) |

**Supplemental Table 5:** Census region–stratified mortality rates (per 100,000) for drug abuse and bladder cancer in the United States, 1999-2023.

| **Year** | **Northeast AAMR (95% CI)** | **Midwest AAMR (95% CI)** | **South AAMR (95% CI)** | **West AAMR (95% CI)** |
| --- | --- | --- | --- | --- |
| 1999 | 0.0742 (0.0526–0.0958) | 0.1201 (0.0839–0.1563) | 0.0742 (0.0526–0.0958) | 0.1353 (0.0969–0.1737) |
| 2000 | 0.0918 (0.0675–0.1161) | 0.1515 (0.1110–0.1920) | 0.1075 (0.0805–0.1345) | 0.1562 (0.1145–0.1979) |
| 2001 | 0.0857 (0.0621–0.1093) | 0.1461 (0.1064–0.1858) | 0.1021 (0.0756–0.1286) | 0.1492 (0.1083–0.1901) |
| 2002 | 0.0804 (0.0572–0.1036) | 0.1486 (0.1088–0.1884) | 0.1039 (0.0772–0.1306) | 0.1448 (0.1044–0.1852) |
| 2003 | 0.3841 (0.3346–0.4336) | 0.6570 (0.5844–0.7296) | 0.3722 (0.3292–0.4152) | 0.4879 (0.4288–0.5470) |
| 2004 | 0.5385 (0.4811–0.5959) | 0.9010 (0.8152–0.9868) | 0.5221 (0.4717–0.5725) | 0.6682 (0.5990–0.7374) |
| 2005 | 0.7248 (0.6582–0.7914) | 1.1899 (1.0888–1.2910) | 0.7025 (0.6451–0.7599) | 0.8593 (0.7805–0.9381) |
| 2006 | 0.8219 (0.7521–0.8917) | 1.2997 (1.1933–1.4061) | 0.7899 (0.7292–0.8506) | 0.9659 (0.8821–1.0497) |
| 2007 | 0.8807 (0.8087–0.9527) | 1.3763 (1.2665–1.4861) | 0.8480 (0.7855–0.9105) | 1.0201 (0.9334–1.1068) |
| 2008 | 1.0116 (0.9343–1.0889) | 1.5308 (1.4153–1.6463) | 0.9862 (0.9191–1.0533) | 1.1240 (1.0325–1.2155) |
| 2009 | 1.0186 (0.9409–1.0963) | 1.5305 (1.4150–1.6460) | 0.9965 (0.9289–1.0641) | 1.1570 (1.0638–1.2502) |
| 2010 | 1.0325 (0.9544–1.1106) | 1.5577 (1.4410–1.6744) | 1.0179 (0.9495–1.0863) | 1.2099 (1.1141–1.3057) |
| 2011 | 1.1083 (1.0281–1.1885) | 1.6759 (1.5557–1.7961) | 1.0913 (1.0209–1.1617) | 1.3052 (1.2060–1.4044) |
| 2012 | 1.2189 (1.1355–1.3023) | 1.8259 (1.7013–1.9505) | 1.2011 (1.1280–1.2742) | 1.4282 (1.3249–1.5315) |
| 2013 | 1.1994 (1.1166–1.2822) | 1.8052 (1.6814–1.9290) | 1.1890 (1.1163–1.2617) | 1.4077 (1.3053–1.5101) |
| 2014 | 1.2265 (1.1430–1.3100) | 1.8334 (1.7088–1.9580) | 1.2199 (1.1463–1.2935) | 1.4259 (1.3230–1.5288) |
| 2015 | 1.2988 (1.2129–1.3847) | 1.9271 (1.7990–2.0552) | 1.2985 (1.2231–1.3739) | 1.5162 (1.4100–1.6224) |
| 2016 | 1.3104 (1.2240–1.3968) | 1.9330 (1.8054–2.0606) | 1.3206 (1.2448–1.3964) | 1.5332 (1.4266–1.6398) |
| 2017 | 1.2696 (1.1848–1.3544) | 1.9081 (1.7815–2.0347) | 1.2969 (1.2218–1.3720) | 1.5062 (1.4006–1.6118) |
| 2018 | 1.2503 (1.1661–1.3345) | 1.8880 (1.7621–2.0139) | 1.2806 (1.2058–1.3554) | 1.4911 (1.3859–1.5963) |
| 2019 | 1.2913 (1.2060–1.3766) | 1.9529 (1.8254–2.0804) | 1.3289 (1.2533–1.4045) | 1.5275 (1.4211–1.6339) |
| 2020 | 1.2414 (1.1577–1.3251) | 1.9057 (1.7793–2.0321) | 1.2817 (1.2070–1.3564) | 1.4810 (1.3754–1.5866) |
| 2021 | 1.2596 (1.1749–1.3443) | 1.9189 (1.7920–2.0458) | 1.3067 (1.2316–1.3818) | 1.5052 (1.3992–1.6112) |
| 2022 | 1.2466 (1.1622–1.3310) | 1.9042 (1.7777–2.0307) | 1.3072 (1.2322–1.3822) | 1.5003 (1.3944–1.6062) |
| 2023 | 1.2262 (1.1422–1.3102) | 1.8801 (1.7542–2.0060) | 1.2850 (1.2104–1.3596) | 1.4752 (1.3697–1.5807) |

**Supplemental Table 6:** State-level death counts (per 100,000) for drug abuse and bladder cancer, United States, 1999–2023.

| **State** | **Deaths (1999–2020)** | **Deaths (2021–2023)** | **Total Deaths (1999–2023)** |
| --- | --- | --- | --- |
| Alaska | 85 | 28 | 113 |
| Arizona | 894 | 312 | 1,206 |
| Arkansas | 352 | 71 | 423 |
| California | 646 | 90 | 736 |
| Colorado | 778 | 259 | 1,037 |
| Connecticut | 761 | 109 | 870 |
| Delaware | 252 | 105 | 357 |
| District of Columbia | 59 | 0 | 59 |
| Florida | 3,410 | 882 | 4,292 |
| Georgia | 715 | 190 | 905 |
| Hawaii | 178 | 38 | 216 |
| Idaho | 547 | 105 | 652 |
| Illinois | 1,686 | 355 | 2,041 |
| Indiana | 1,324 | 311 | 1,635 |
| Iowa | 709 | 196 | 905 |
| Kansas | 689 | 152 | 841 |
| Kentucky | 899 | 265 | 1,164 |
| Louisiana | 516 | 137 | 653 |
| Maine | 402 | 152 | 554 |
| Maryland | 1,156 | 208 | 1,364 |
| Massachusetts | 551 | 257 | 808 |
| Michigan | 2,797 | 607 | 3,404 |
| Minnesota | 1,058 | 375 | 1,433 |
| Mississippi | 82 | 36 | 118 |
| Missouri | 1,020 | 328 | 1,348 |
| Montana | 419 | 89 | 508 |
| Nebraska | 626 | 120 | 746 |
| Nevada | 406 | 137 | 543 |
| New Hampshire | 507 | 88 | 595 |
| New Jersey | 2,059 | 343 | 2,402 |
| New Mexico | 347 | 72 | 419 |
| New York | 3,785 | 654 | 4,439 |
| North Carolina | 1,051 | 401 | 1,452 |
| North Dakota | 294 | 41 | 335 |
| Ohio | 2,960 | 659 | 3,619 |
| Oklahoma | 664 | 134 | 798 |
| Oregon | 1,827 | 388 | 2,215 |
| Pennsylvania | 2,632 | 500 | 3,132 |
| Rhode Island | 333 | 57 | 390 |
| South Carolina | 833 | 192 | 1,025 |
| South Dakota | 285 | 56 | 341 |
| Tennessee | 817 | 331 | 1,148 |
| Texas | 3,682 | 900 | 4,582 |
| Utah | 309 | 66 | 375 |
| Vermont | 339 | 60 | 399 |
| Virginia | 522 | 305 | 827 |
| Washington | 2,231 | 478 | 2,709 |
| West Virginia | 163 | 105 | 268 |
| Wisconsin | 2,055 | 331 | 2,386 |
| Wyoming | 191 | 54 | 245 |
| **Total** | **51,057** | **12,193** | **63,250** |

**Supplemental Table 7:** State-level death counts for drug abuse and bladder cancer, United States, 1999–2023.

| **State** | **Deaths (1999–2020)** | **Deaths (2021–2023)** | **Deaths (1999–2023)** | **% of Total Deaths (1999–2023)** |
| --- | --- | --- | --- | --- |
| Alabama | 154 | 64 | 218 | 0.34% |
| Alaska | 85 | 28 | 113 | 0.18% |
| Arizona | 894 | 312 | 1,206 | 1.91% |
| Arkansas | 352 | 71 | 423 | 0.67% |
| California | 646 | 90 | 736 | 1.16% |
| Colorado | 778 | 259 | 1,037 | 1.64% |
| Connecticut | 761 | 109 | 870 | 1.38% |
| Delaware | 252 | 105 | 357 | 0.56% |
| District of Columbia | 59 | 0 | 59 | 0.09% |
| Florida | 3,410 | 882 | 4,292 | 6.79% |
| Georgia | 715 | 190 | 905 | 1.43% |
| Hawaii | 178 | 38 | 216 | 0.34% |
| Idaho | 547 | 105 | 652 | 1.03% |
| Illinois | 1,686 | 355 | 2,041 | 3.23% |
| Indiana | 1,324 | 311 | 1,635 | 2.58% |
| Iowa | 709 | 196 | 905 | 1.43% |
| Kansas | 689 | 152 | 841 | 1.33% |
| Kentucky | 899 | 265 | 1,164 | 1.84% |
| Louisiana | 516 | 137 | 653 | 1.03% |
| Maine | 402 | 152 | 554 | 0.88% |
| Maryland | 1,156 | 208 | 1,364 | 2.16% |
| Massachusetts | 551 | 257 | 808 | 1.28% |
| Michigan | 2,797 | 607 | 3,404 | 5.38% |
| Minnesota | 1,058 | 375 | 1,433 | 2.27% |
| Mississippi | 82 | 36 | 118 | 0.19% |
| Missouri | 1,020 | 328 | 1,348 | 2.13% |
| Montana | 419 | 89 | 508 | 0.80% |
| Nebraska | 626 | 120 | 746 | 1.18% |
| Nevada | 406 | 137 | 543 | 0.86% |
| New Hampshire | 507 | 88 | 595 | 0.94% |
| New Jersey | 2,059 | 343 | 2,402 | 3.80% |
| New Mexico | 347 | 72 | 419 | 0.66% |
| New York | 3,785 | 654 | 4,439 | 7.02% |
| North Carolina | 1,051 | 401 | 1,452 | 2.30% |
| North Dakota | 294 | 41 | 335 | 0.53% |
| Ohio | 2,960 | 659 | 3,619 | 5.72% |
| Oklahoma | 664 | 134 | 798 | 1.26% |
| Oregon | 1,827 | 388 | 2,215 | 3.50% |
| Pennsylvania | 2,632 | 500 | 3,132 | 4.95% |
| Rhode Island | 333 | 57 | 390 | 0.62% |
| South Carolina | 833 | 192 | 1,025 | 1.62% |
| South Dakota | 285 | 56 | 341 | 0.54% |
| Tennessee | 817 | 331 | 1,148 | 1.82% |
| Texas | 3,682 | 900 | 4,582 | 7.24% |
| Utah | 309 | 66 | 375 | 0.59% |
| Vermont | 339 | 60 | 399 | 0.63% |
| Virginia | 522 | 305 | 827 | 1.31% |
| Washington | 2,231 | 478 | 2,709 | 4.28% |
| West Virginia | 163 | 105 | 268 | 0.42% |
| Wisconsin | 2,055 | 331 | 2,386 | 3.77% |
| Wyoming | 191 | 54 | 245 | 0.39% |
| **Total** | **51,057** | **12,193** | **63,250** | **100.00%** |

**Supplemental Table 8:** Percentage distribution of place of death for drug abuse and bladder cancer fatalities, United States, 1999–2023.

| **Place of Death** | **Deaths (1999–2020)** | **Deaths (2021–2023)** | **Total Deaths (1999–2023)** | **% of Total Deaths** |
| --- | --- | --- | --- | --- |
| Medical Facility – Inpatient | 11,876 | 2,197 | 14,073 | 22.56% |
| Medical Facility – Outpatient or ER | 1,120 | 276 | 1,396 | 2.24% |
| Medical Facility – Dead on Arrival | 79 | 0 | 79 | 0.13% |
| Decedent's home | 22,835 | 6,487 | 29,322 | 47.01% |
| Hospice facility | 5,686 | 1,414 | 7,100 | 11.38% |
| Nursing home / long term care | 7,060 | 1,367 | 8,427 | 13.51% |
| Other | 2,363 | 454 | 2,817 | 4.52% |
| Place of death unknown | 37 | 0 | 37 | 0.06% |
| **Total** | **51,056** | **12,195** | **63,251** | **100.00%** |

**Supplementary Figure 1:** State stratified age-adjusted mortality rates per 100,000 for drug abuse and bladder cancer fatalities related mortality in United States, 1999-2023.

**Supplementary Figure 2:** Percentage distribution of place of death in the United States (1999–2023), for drug abuse and bladder cancer fatalities related mortality highlighted.
